# Protein Structures among Bio-Ethanol Co-Products and Its Relationships with Ruminal and Intestinal Availability of Protein in Dairy Cattle

**DOI:** 10.3390/ijms140816802

**Published:** 2013-08-15

**Authors:** Arash Azarfar, Arjan Jonker, Peiqiang Yu

**Affiliations:** 1 Department of Animal and Poultry Science, College of Agriculture and Bio-Resources, University of Saskatchewan, Saskatoon, SK S7N 5A8, Canada; E-Mails: arash.azarfar@gmail.com (A.A.); arjan.jonker@agresearch.co.nz (A.J.); 2 Faculty of Agriculture, Lorestan University, PO Box 465, Khorramabad, Iran; 3 Grasslands Research Centre, AgResearch Ltd., Tennent Drive, Private Bag 11008, Palmerston North 4442, New Zealand; 4 Department of Animal Science, Tianjin Agricultural University, 22 Jinjin Road, Xiqing District, Tianjin 300384, China

**Keywords:** protein molecular structures, α-helix to β-sheet ratio, dried distillers grains with soluble (DDGS), intestinal digestibility

## Abstract

The objectives of this study were to reveal molecular structures of protein among different types of the dried distillers grains with solubles (100% wheat DDGS (WDDGS); DDGS blend1 (BDDGS1, corn to wheat ratio 30:70%); DDGS blend2 (BDDGS2, corn to wheat ratio 50:50 percent)) and different batches within DDGS type using diffuse reflectance infrared Fourier transform spectroscopy (DRIFT). Compared with BDDGS1 and BDDGS2, wheat DDGS had higher (*p* < 0.05) peak area intensities of protein amide I and II and amide I to II intensity ratio. Increasing the corn to wheat ratio form 30:70 to 50:50 in the blend DDGS did not affect amide I and II area intensities and their ratio. Amide I to II peak intensity ratio differed (*p* < 0.05) among the different batches within WDDGS and BDDGS1. Compared with both blend DDGS types, WDDGS had higher α-helix and β-sheet ratio (*p* < 0.05), while α-helix to β-sheet ratio was similar among the three DDGS types. The α-helix to β-sheet ratio differed significantly among batches within WDDGS. Principal component analysis (PCA) revealed that protein molecular structures in WDDGS differed from those of BDDGS1 and between different batches within BDDGS1 and BDDGS2. The α-helix to β-sheet ratios of protein in all DDGS types had an influence on availability of protein at the ruminal level as well as at the intestinal level. The α-helix to β-sheet ratio was positively correlated to rumen undegraded protein (*r* = 0.41, *p* < 0.05) and unavailable protein (PC; *r* = 0.59, *p* < 0.05).

## 1. Introduction

The conventional method to determine the nutrient make-up of feeds is the traditional “wet” chemical analysis. This so-called “wet” chemical analysis gives no information on inherent molecular structure of nutrients and structural matrices of feeds. The molecular structures of nutrients, including proteins, are associated with their accessibility for gastrointestinal digestive enzymes and with their digestive behavior [1]. As opposed to “wet” chemical analysis, infrared spectroscopic techniques, such as Diffuse Reflectance Infrared Fourier Transform Spectroscopy (DRIFT), have the potential to reveal molecular structures of proteins. These techniques have been implemented to study the protein secondary structure of feeds [2,3].

Co-products from bio-ethanol processing such as dried distillers grains with solubles (DDGS) have been utilized as protein supplements in both dairy and beef cattle rations. Despite being excellent sources of intestinally digestible and absorbable protein [4,5], concerns have arisen with regard to inconsistency of nutrient profile of these co-products as it may lead to formulation of imbalanced diets for ruminants. The bio-ethanol plant-to-plant variations in nutrient content and nutrient availability of DDGS is documented [4,6]. The objectives of this study were to reveal the molecular structures of protein among different types of DDGS and different batches within each DDGS type using DRIFT, and to investigate if secondary structures of protein correlate to ruminal degradability and intestinal digestibility of protein in dairy cattle estimated by the approach of van Duinkerken *et al.* [7].

## 2. Results and Discussion

### 2.1. Protein Molecular Structures, Amide I and Amide II and Their Ratio among Different Types and Batches of DDGS

**Figure 1:**
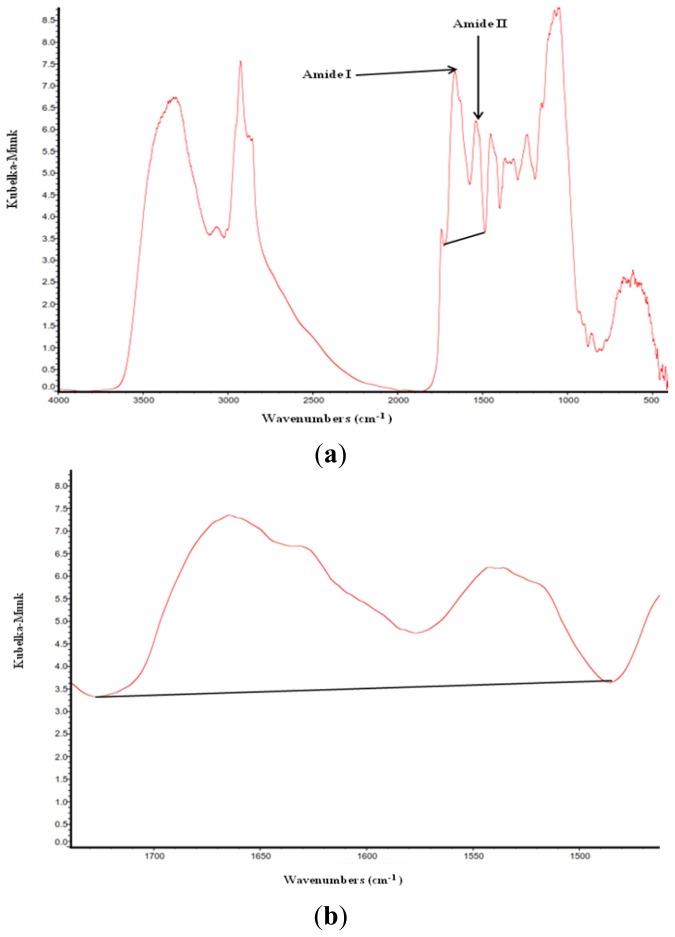
Typical full-range spectrum of DDGS with (**a**) peak area of amide I and amide II (at *ca*. 1719–1485 cm^−1^) and (**b**) enlargement of amide I and II area.

Recently, several studies determined the relationship between protein molecular structures (amide I, amide II and their ratios) and the nutritive value of several feedstuffs [1–3,8]. The spectrum of protein in all DDGS samples and batches had two primary features being the amide I (at *ca.* 1665 cm^−1^) and amide II (at *ca.* 1550 cm^−1^) in the spectra regions of *ca.* 1719–1485 cm^−1^ (Figure 1a). These two features are frequently used to assess protein conformation [9,10]. Compared with BDDGS1 (blend DDGS, wheat to corn ratio 70:30) and BDDGS2 (blend DDGS, wheat to corn ratio 50:50), the WDDGS (wheat DDGS) had higher (*p* < 0.05) peak area intensities of protein amide I and II and amide I to amide II peak intensity ratio (Table 1). The results are consistent with the findings of Yu *et al.* [3] who found that WDDGS had a higher amide I to II peak intensity ratio compared with blend DDGS. The results indicated that increasing the corn to wheat ratio from 30%:70% to 50%:50% in the blend DDGS did not affect protein amide I and II peak area intensities and their ratio (Table 1).

The different batches within WDDGS and BDDGS1 had different amide I to II peak intensity ratios (Table 2), which indicates a difference in protein values for dairy cattle from different batches within WDDGS and BDDGS1. Indeed, rumen undegraded protein (RUP) significantly differed among different batches within WDDGS (420.0, 378.0 and 330.6 g/kg crude protein (CP) in batch 1, batch 2 and batch 3, respectively) and BDDGS1 (673.5 and 511.8 g/kg CP in batch 1 and batch 2, respectively) [5], which may be due to the differences in their protein molecular structures.

### 2.2. Protein Secondary Structure Profile and Ratio among Different Types and Batches of DDGS

Despite methodological considerations that have to be taken into account when using α-helix to β-sheet ratio to study the secondary structure of proteins [11], this criterion has been successfully used to explore differences in protein secondary structure of feedstuffs [8,12].

The protein amide I peak centered at 1663 cm^−1^ for α-helix and 1632 cm^−1^ for β-sheet in different co-products and batches of different bio-ethanol processing plants (Tables 3 and 4). These results were in the range of those reported by Yu *et al.* [3]. The α-helix and β-sheet intensities were significantly higher in WDDGS than in BDDGS1 and BDDGS2 (Table 3), while α-helix to β-sheet ratio was similar among the different types of DDGS. A high proportion of β-sheet in the secondary structure of proteins is thought to decrease access of gastrointestinal digestive enzymes to break down the protein structures, which in turn leads to a low protein value for the animal [13]. Therefore, the similar α-helix to β-sheet ratio among DDGS types may explain why the DVE (true protein digested and absorbed in the small intestine) supply in dairy cows was similar among the DDGS types (Table 5).

The intensity of the peak height of α-helix and β-sheet were similar between the different batches within BDDGS1 and BDDGS2, while α-helix to β-sheet ratios significantly differed among the batches within WDDGS (Table 4; *p* < 0.05).

### 2.3. Multivariate Analysis of Spectra from Protein Internal Structures among Different Types and Batches of DDGS

The spectra of protein molecular structures, in the region ca. 1719–1485 cm^−1^, analyzed by cluster analysis (CLA) has been used to discriminate the molecular structural differences among feedstuffs [3,12]. The CLA performs an agglomerative hierarchical cluster analysis of a spectral data set and displays the results as a dendogram. First it calculates a distance matrix, which contains information on the similarity of the spectra. Then, by hierarchical clustering, the algorithm searches within the distance matrix for the two most similar spectra (minimal distance). These spectra are combined into a new object (a “cluster” or “hierarchical group”). The spectral distances between all remaining spectra and the new cluster are then recalculated [14].

**Figure 2:**
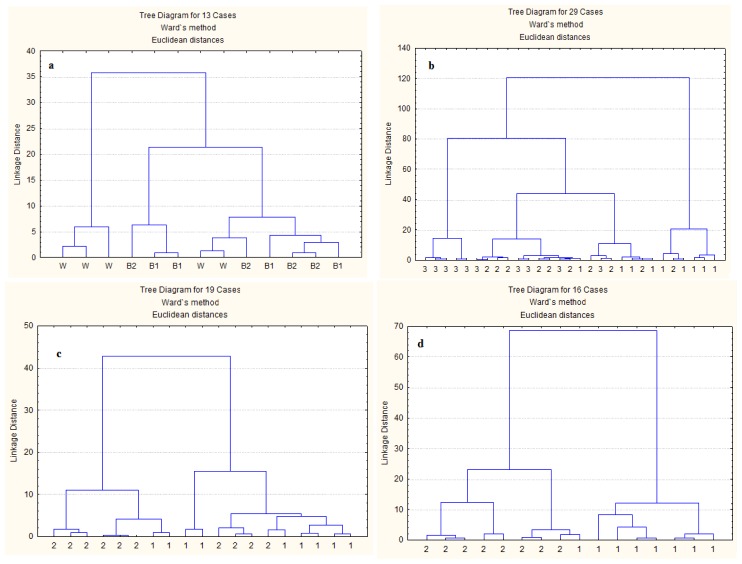
Cluster analysis of protein molecular spectra: (**a**) Wheat DDGS (W), BDDGS1 (B1; 30% corn and 70% wheat) and BDDGS2 (B2, 50% corn and 50% wheat); (**b**) Three batches within wheat DDGS; (**c**) Two batches within BDDGS1; and (**d**) Two batches within BDDGS2.

The mixed dendrogram (Figure 2a) of WDDGS, BDDGS1 and BDDGS2 showed similarity of spectral data in their amide I and II regions, indicating that they were not completely different in protein spectroscopic features. Liu *et al.* [15] could also not separate spectra in the amide I and II region of wheat DDGS from corn DDGS by CLA. Four classes were distinguished below linkage distance less than 19 with CLA for different batches within WDDGS (Figure 2b). All cases of batch 3 were in class 1 and 2, except for one case in class 3, all cases of batch 1 were in class 3 and 4, except for one case in class 2, and all cases of batch 2 were in class 2 and 3, except for one case in class 4. Therefore, batch 1 and 3 were almost completely separated while there was a larger overlap of batch 2 with both batch 1 and 3. Gamage *et al.* [16] performed CLA on the fingerprint region (1800–800 cm^−1^) of three batches of WDDGS and they could separate two batches from each other with the third batch having overlap with both other batches.

The spectra from two batches within BDDGS1 formed three distinct classes just below linkage distance of 18 (Figure 2c). The cases of both batches were spread over the classes, which suggest that the protein spectral features were similar for both batches.

The spectra for two batches within BDDGS2 were grouped into two distinct clusters below the linkage distance of 28, except for one spectrum of batch 1 (Figure 2d), indicating that protein spectral features were different for batch 1 and 2.

The second multivariate analysis used in the current study was principal component analysis (PCA), which is a statistical data reduction method. The PCA was used to identify similarities or variations in the protein amide I and amide II spectra of the different DDGS types and their different batches.

**Figure 3:**
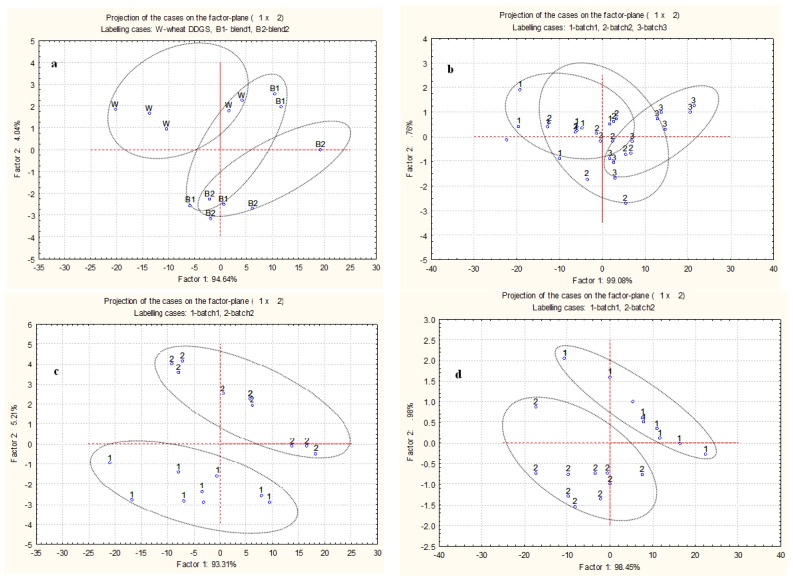
Principal component analysis with 1st *vs.* 2nd principal component of spectra from protein molecular structures in: (**a**) Wheat DDGS (W), BDDGS1 (B1; 30% corn and 70% wheat) and BDDGS2 (B2, 50% corn and 50% wheat); (**b**) Three batches within wheat DDGS; (**c**) Two batches within BDDGS1; and (**d**) Two batches within BDDGS2.

Protein molecular structures of WDDGS were discriminated by PCA from BDDGS2 into separate ellipses, while the protein molecular structures of BDDGS2 were similar to both WDDGS and BDDGS1 (Figure 3a).

The ellipses of batches 1 and 3 in WDDGS were almost completely separated, while the ellipse of batch 2 overlapped with the ellipses of the other two batches (Figure 3b). A similar trend was seen for the fingerprint region of three batches of WDDGS [16]. The inherent amide I and II protein structures differed among the batches of BDDGS1 and BDDGS2 as they were grouped into two separate ellipses with no overlapping (Figure 3c,d). This confirms the results of CLA that molecular structures of protein in a specific type of DDGS may differ between its different batches.

### 2.4. Correlation between α-Helix to β-Sheet Ratios and Nutrient Profile

The α-helix to β-sheet ratio correlated with intermediately degradable true protein (PB2; *r* = −0.40, *p* = 0.035), unavailable protein (PC; *r* = 0.59, *p* = 0.001), and RUP (*r* = 0.41, *p* = 0.031) and tended to correlate with rumen degraded protein balance (OEB; *r* = −0.34, *p* = 0.080; Table 6). The positive correlation with RUP and negative correlation with OEB is consistent with Liu *et al.* [15]. The α-helix to β-sheet ratio did not correlate with intestinal digestibility of RUP *in vitro* and DVE (Table 6). In Yu and Nuez-Ortín [17], α-helix to β-sheet ratio positively correlated with protein fraction PC. The PC fraction was undegradable, and contained proteins associated with lignin and tannins and heat-damaged proteins [17]. These results indicate that a higher α-helix to β-sheet ratio may result in a higher undegradable protein content in the DDGS. There was a positive correlation between α-helix to β-sheet ratio and intestinal digestibility of RUP *in vitro*, but it was not significant (Table 6). This result was opposite to the previous findings of Liu *et al.* [15] and Yu and Nuez-Ortín [17]. They found that α-helix to β-sheet ratio negatively correlated to intestinal digestibility of RUP *in vitro*. We do not have a plausible explanation for such a discrepancy.

The results of this study indicated that at least for the different types of DDGS, the ratio of α-helix to β-sheet in proteins had an influence on the availability of protein in the rumen (PB2 and OEB) and intestine (PC and RUP) in dairy cattle.

## 3. Experimental Section

### 3.1. Sample Collection and Preparation

From February 2009 to January 2010, seven different batches of wheat DDGS and blend DDGS (BDDGS1, wheat to corn ratio 70:30; BDDGS2, wheat to corn ratio 50:50) were collected from two bio-ethanol processing plants located in Saskatchewan, Canada. Three different batches of wheat DDGS were sampled on December 13 and 27 of 2009 and January 11 of 2010. Batches of blend DDGS (two batches for each blend) were collected on February 6 (blend2), February 9 (blend1), February 17 (blend1) and March 9 (blend2) of 2009. On a given sampling day, three samples of DDGS were taken over a period of 24 h. However, only two samples were used for wet chemical profiling, *in situ* rumen studies and determination of protein molecular structures.

### 3.2. Diffused Reflectance Fourier Transformed Infrared Spectroscopy (Drift)

The DDGS samples were two times finely ground to pass through a 0.25 mm screen (Retsch ZM-1, Brinkmann Instruments LTD, Mississauga, ON, Canada). Samples of ground DDGS were then mixed with KBr in a ratio of one part of co-product with four parts of KBr in a 2 mL centrifuge tube and vortexed for 10 s. Diffuse reflectance infrared Fourier transform spectroscopy was performed using a Bio-Rad FTS-40 with a ceramic IR source and MCT detector (Bio-Rad laboratories, Hercules, CA, USA). Data was collected using Win-IR software. Spectra were generated from the mid-IR (4000–800 cm^−1^) portion of electromagnetic spectrum with 256 co-added scans and a spectral resolution of 4 cm^−1^ (Figures 1a). Spectral analysis was done with OMNIC 7.2 software (Spectra Tech., Madison, WI, USA, 2006). Protein amides I and II and protein secondary structures α-helix and β-sheet were identified according to the published reports [18,19].

### 3.3. Amide I, Amide II and α-Helix and β-Sheet Ratio Identification

The amide I and amide II absorption intensity of the peak area and their ratio were calculated with baseline *ca*. 1719–1485 cm^−1^ (Figure 3a,b). The protein amide I bond is primarily a C=O stretching vibration (80%) plus C–N stretching vibration which absorbs at ca. 1655 cm^−1^ [9,20]. Protein amide II consists primarily of N–H bending vibrations (60%) along with C–N stretching vibrations (40%) absorbs at *ca.* 1550 cm^−1^ [9,20]. Protein secondary structures were determined using the amide I functional group band located in the region of ca. 1719–1576 cm^−1^. The intensity of the peak height at *ca.* 1663 (α-helix) and 1632 (β-sheet) cm^−1^ was used to calculate α-helix to β-sheet ratio.

### 3.4. Chemical Analysis and Protein Partitioning

The detailed chemical analysis, protein partitioning and chemical profiles were described by Azarfar *et al.* [21].

### 3.5. Rumen Incubation Procedure

Four rumen fistulated (internal diameter 10cm; Bar Diamond Inc., Parma, OH, USA) non-pregnant dry Holstein Frisian cows were used in an *in situ* trail, which had been reviewed and approved by Animal Care Committee of the University of Saskatchewan (Animal Use Protocol # 19910012). The cows were individually housed in pens at the experimental farm of the University of Saskatchewan (Saskatoon, SK, Canada) and cared for according the Canadian Council on Animal Care guidelines [22]. The cows had free access to water and were fed 15 kg DM/day total mixed ration twice daily in equal portions at 8.00 a.m. and 4.00 p.m. The total mixed ration consisted in %DM of 55% barley silage, 12.5% alfalfa hay, 5% dehydrated alfalfa and 27.5% concentrates as described in Yu *et al.* [23].

In order to generate a homogeneous sample, DDGS samples were processed using a laboratory scale roller mill (gap, 0.203 mm; Apollo Machine and Products Ltd, Saskatoon, SK, Canada) prior to the *in situ* trial. *In situ* rumen degradation kinetics were determined as described by Yu *et al.* [24]. Approximately seven grams of sample were weighed into pre-weighed numbered nylon bags (10 × 20 cm; pore size of 41 μm; Nitex 03–41/31 monofilament open mesh fabric, Screentec Corp., Mississauga, ON, Canada), resulting in a sample-to-bag surface ratio of 17.5 mg/cm^2^. The bags were randomly assigned to the four cows, and incubated in the rumen in two runs for 72, 36, 12, 6 and 2 h by the “all-out method”. A polyester mesh bag (45 cm × 45 cm with a 90 cm length of rope to be anchored to the cannula) was used to hold the bags in the rumen. For incubation times of 72 h, 36 h, 12 h, 6 h and 2 h, seven, six, five, four and two bags of each sample were randomly incubated in the rumen of each cow, respectively. Immediately after retrieval, all bags were placed in cold tap water to stop microbial fermentation and then washed manually five times in cold tap water followed by oven drying at 55 °C for 48 h. The 0 h incubation samples were washed by the procedure described by Azarfar *et al.* [25] to fractionated washable fraction (W) into a truly washable soluble fraction (S) and a washable but insoluble fraction (WI). Since the WI was almost zero, for the purpose of modelling it was assumed that the W fraction equalled the S fraction. Incubation residue from the treatment bags were pooled within time and incubation run.

### 3.6. Rumen Degradation Kinetics

Protein and carbohydrate were fractionated using *in situ* approach described in the updated version of the DVE/OEB protein evaluation system [7], in which, CP, neutral detergent fibre (NDF), and residual non-starch polysaccharide (RNSP; calculated as OM − (CP + CFat + starch + ESC + NDF)) were partitioned into a truly soluble fraction (S), a washable but insoluble fraction (WI), a non-washable potentially degradable fraction [D; calculated as (1000 − (S + WI) − U)] and a non-washable undegradable fraction (U; the residue after 72 h of rumen incubation). In the current study WI of CP was assumed to be zero as described earlier. The ESC is designated S fraction of carbohydrates, and was assumed to degrade instantly in the rumen [7]. The rest of carbohydrates were classified into NDF, RNSP and starch. The washable RNSP and starch (WI_RNSP_ and WI_Starch_, respectively) were assumed to contain only insoluble material, while NDF was assumed to contain no washable fraction. Since the incubation residues were not directly analyzed for CFat, the correction factors 65%, 44%, 17% and 3% of the original CFat were applied to calculate RNSP for the 0 h, 2 h, 6 h and 12 h incubation residues, respectively [7].

Fractional degradation rates of D for CP, NDF and RNSP (*Kd*,/h) were calculated by fitting the degradation data to a first-order kinetics model described by Robinson *et al.* [26]:

(1) $$R(t)=U+D\times {\text{e}}^{-Kd\times (t-\text{lag})}$$

where *R*(*t*) is residue (g/kg) left after t hours of rumen incubation (h), lag is lag time (h) and *Kd* is fractional degradation rate of *D* fraction (/h).

Since the *U* and lag are assumed to be zero for starch, the following model was applied to estimate *Kd* of *D* fraction for starch [27]:

(2) $$R(t)=(100-W)\times {\text{e}}^{-Kd\times t}$$

where *W* is washable fraction. The parameters of models were calculated using the NLIN procedure of SAS 9.2 [28] with iterative least squares regression (Gauss-Newton method).

Due to the lack of fit, effective rumen degradation was not determined for RNSP of BDDGS1 and BDDGS2.

### 3.7. Intestinal Digestion of Rumen Undegraded Protein

The estimation of intestinal digestion was determined by two methods. In the first method intestinal digestibility of rumen undegraded protein (RUP) was calculated as:

(3) $$\text{dRUP}=(\text{RUP}-U_{\text{CP}})/\text{RUP}\times 1000$$

where dRUP is digestibility of RUP (g/kg RUP) and *U*_CP_ is undegraded fraction of CP after 72 h rumen incubation. In the second method a three steps *in vitro* approach as described by Calsamiglia and Stern [29] was used to estimate dRUP.

### 3.8. The DVE/OEB Protein Evaluation System

The protein value of DDGS for cattle was evaluated with the DVE/OEB protein evaluation system [7]. Methodology and full results are described in Azarfar *et al.* [5].

### 3.9. Statistical Analysis

Statistical analyses were performed using the MIXED procedure of SAS 9.2 [28]. The models used were:

(4) $$Y_{ij}=\mu +F_{i}+e_{ij}$$

(5) $$Y_{ij}=\mu +B_{i}+e_{ij}$$

where, *Yij* is an observation of the dependent variable *ij* (amide I, amide II, α-helix, β-sheet and their ratios); *μ* is the population mean for the variable; *F_i_* is the fixed effect of feed sources (*i* = 3; WDDGS, BDDGS1 and BDDGS2); *B_i_* is the fixed effect of batch (*i* = 2 for WDDGS and 3 for BDDGS1 and BDDGS2) and *e_ij_* is the random error associated with the observation *ij*. Model 1 was used to study the effects of feed sources, while the second model was used to compare the different batches within DDGS source. In the first model batch and sample were regarded as experimental replicates whereas in the second model sample was considered as replicate.

For all statistical analyses, significance was declared at *p* < 0.05. The Fisher’s protected least significant difference (LSD) test was used for multiple treatment comparisons using the LSMEANS of SAS 9.2 [28] with letter grouping obtained using SAS pdmix800 macro [30].

Relationship between the ratio of α-helix to β-sheet with protein sub-fractions, rumen undegraded protein (RUP), intestinal digestibility of RUP, true protein digested and absorbed in the small intestine (DVE), and rumen degraded protein balance were performed using the CORR procedure of SAS 9.2 [28] using a Pearson correlation method. The normality check was performed using the UNIVARIATE Procedure of SAS 9.2 [28].

### 3.10. Multivariate Analysis of Drift Protein Molecular Spectra

Agglomerated hierarchical cluster analysis (CLA) and principal component analysis (PCA) were performed using Statistica software 9.0 (StatSoft Inc., Tulsa, OK, USA, 2007) to classify and distinguish the inherent differences of protein molecular structures among the DDGS samples and batches within DDGS source. Spectral region 1719–1485 cm^−1^ was used for CLA and PCA. For the CLA, Ward’s algorithm method was used without any prior parameterization of spectral data. For the PCA, the first two principal components were plotted.

## 4. Conclusions

The results of this study indicate that dried distillers grains with solubles are good sources of intestinally digested and absorbed protein for ruminants. However, the variation in protein molecular structures among batches within DDGS and therefore their protein values are factors that have to be taken into account before their inclusion in ruminant diets. The cluster and principal component analyses reveal that the protein molecular structures in a specific type of DDGS may differ between different batches and that these differences are easily revealed by univariate and multivariate analysis of DRIFT spectra in the molecular protein region. The results of the current study indicate that protein secondary structures, α-helix to β-sheet ratio, were significantly correlated with rumen undegraded protein and tended to have a significant correlation with rumen degraded protein balance. Therefore, the secondary structures of protein affect the protein supply to dairy cattle.

## Figures and Tables

**Table 1:** Amide I and II and their ratios: Comparison of wheat DDGS and two types of blend DDGS using DRIFT molecular spectroscopy.

Items	Number of repetitions for DRIFT analysis	Amide I	Amide II	Ratio of amide I to amide II
Amides IR peak center position		~1655 cm^−1^	~1550 cm^−1^	~1665/~1550 cm^−1^
Amides IR peak region		~1719–1576 cm^−1^	~1576–1450 cm^−1^	
Amides IR peak are base line		~1719–1485 cm^−1^	~1719–1485 cm^−1^	~1719–1485 cm^−1^

		**Based on the protein amide I and II peak area**		

WDDGS 1	30	220.13 a	99.40 a	2.18 a
BDDGS1 2	20	106.66 b	57.62 b	1.90 b
BDDGS2 3	20	83.29 b	44.92 b	1.83 b
SEM 4		27.380	11.364	0.624

a,b Means with different letters in the same column are significantly different (*p* < 0.05). Multi-treatment comparison method: LSD.

1 Wheat DDGS;

2 Blend DDGS (corn to wheat ratio 30%:70%);

3 Blend DDGS (corn to wheat ratio 50%:50%);

4 SEM = standard error of the mean.

**Table 2:** Amide I and II and their ratios: Comparison of different batches within wheat DDGS and two types of blend DDGS using DRIFT molecular spectroscopy.

Items	Number of repetitions for DRIFT analysis	Amide I	Amide II	Ratio of amide I to amide II
Amides IR peak center position		~1655 cm^−1^	~1550 cm^−1^	~1665/~1550 cm^−1^
Amides IR peak region		~1719–1576 cm^−1^	~1576–1450 cm^−1^	
Amides IR peak are base line		~1719–1485 cm^−1^	~1719–1485 cm^−1^	~1719–1485 cm^−1^

**WDDGS**		**Based on the amide I and II peak area**		

Batch1	10	312.76	136.05	2.29 a
Batch2	10	200.20	92.59	2.15 a,b
Batch3	10	147.42	65.56	2.11^b^
SEM		39.237	15.526	0.033

**BDDGS1**				

Batch1	10	117.14	58.79	1.73^a^
Batch2	10	102.17	56.44	2.07^b^
SEM	10	8.884	4.513	0.022

**BDDGS2**				

Batch1	10	66.32	37.68	1.74
Batch2	10	100.27	52.30	1.91
SEM	10.145	4.608	0.039	

a,b Means with the different letters in the same column are significantly different (*p* < 0.05). Multi-treatment comparison method: LSD. Abbreviations are explained in Table 1.

**Table 3:** Characteristics of protein secondary structures (α-helix, β-sheet, and their ratio): Comparison of wheat DDGS and two types of blend DDGS using DRIFT molecular spectroscopy.

Infrared absorption					Protein secondary structures		

					α-helix	β-sheet	Ratio of α-helix to β-sheet
Items	Number of repetition for DRIFT analysis	α-helix peak center (cm^−1^)	β-sheet peak center (cm^−1^)	peak base line (cm^−1^)	~1663 (cm^−1^)	~1632 (cm^−1^)	~1663/1632 (cm^−1^)

					Based on the protein α-helix and β-sheet peak height		

WDDGS	30	1665	1632	~1718–1485	2.54 a	2.03 a	1.26
BDDGS1	20	1663	1632	~1718–1485	1.36 b	1.11 b	1.23
BDDGS2	20	1663	1632	~1718–1485	1.10 b	0.85 b	1.29
SEM					0.325	0.260	0.022

a,b Means with different letters in the same column are significantly different (*p* < 0.05). Multi-treatment comparison method: LSD. Abbreviations are explained in Table 1.

**Table 4:** Characteristics of protein secondary structures (α-helix, β-sheet, and their ratio): Comparison of different batches within wheat DDGS and two types of blend DDGS using DRIFT molecular spectroscopy.

Infrared absorption					Protein secondary structures		

					α-helix	β-sheet	Ratio of α-helix to β-sheet
Items	Number of repetition for DRIFT analysis	α-helix peak center (cm^−1^)	β-sheet peak center (cm^−1^)	peak base line (cm^−1^)	~1663 (cm^−1^)	~1632 (cm^−1^)	~1663/1632 (cm^−1^)

**WDDGS**					Based on the protein α-helix and β-sheet peak height		

Batch1	10	1664	1632	~1718–1485	3.60	2.89	1.25 b
Batch2	10	1666	1632	~1718–1485	2.34	1.82	1.29 a
Batch3	10	1665	1632	~1718–1485	1.71	1.38	1.23 b
SEM					0.465	0.371	0.006

**BDDGS1**							

Batch1	10	1664	1632	~1718–1485	1.36	1.06	1.28
Batch2	10	1661	1632	~1718–1485	1.36	1.16	1.17
SEM					0.124	0.078	0.030

**BDDGS2**							

Batch1	10	1662	1632	~1718–1485	0.96	0.74	1.31
Batch2	10	1665	1632	~1718–1485	1.23	0.97	1.28
SEM					0.143	0.095	0.027

a,b Means with the different letters in the same column are significantly different (*p* < 0.05). Multi-treatment comparison method: LSD. Abbreviations are explained in Table 1.

**Table 5:** Rumen undegraded protein, protein digestibility and digested rumen undegraded protein in wheat and blend DDGS.

Items	WDDGS	BDDGS1	BDDGS2	SEM
Rumen undegraded protein (RUP, g/kg CP)	376.2 b	592.7 a	593.9 a	20.69
Rumen degraded protein balance (OEB, g/kg DM)	159.1 a	82.0 b	65.8 b	8.25
*In vitro* intestinal digestibility 1 of RUP (g/kg RUP)	806.9	781.5	788.0	15.03
True protein digested and absorbed in the small intestine (DVE, g/kg DM)	177.7	184.8	170.4	5.00

SEM = standard error of mean.

a,b Means with the different letters in the same row are significantly different (*p* < 0.05). Multi-treatment comparison method: LSD. Abbreviations are explained in Table 1.

1 Intestinal digestion of rumen undegraded protein estimated using the tree step *in vitro* assay.

**Table 6:** Correlation between α-helix to β-sheet ratios and protein sub-fractions, and ruminal and intestinal availability of protein based on the DVE/OEB 2010 protein evaluation system in the different types of DDGS.

Items	Correlation with protein α-helix to β-sheet ratios	

	Correlation coefficient, *r*	*p-*value
**Protein fraction**^1^**(g/kg DM)**		
PA	−0.21	0.280
PB1	−0.06	0.765
PB2	−0.40	0.035
PB3	0.24	0.219
PC	0.59	0.001

Ruminal and intestinal availability of protein		
Rumen undegraded protein (RUP, g/kg CP)	0.41	0.031
Rumen degraded protein balance (OEB, g/kg DM)	−0.34	0.080
*In vitro* intestinal digestibility of RUP (g/kg RUP)	0.10	0.615
True protein digested and absorbed in the small intestine (DVE, g/kg DM)	0.15	0.448

1 PA = non-protein nitrogen; PB1 = soluble true protein; PB2 = intermediately degradable true protein; PB3 = slowly degradable true protein; PC = indigestible protein.

## Abbreviations

CFat: crude fat
CP: crude protein
DM: dry matter
DVE: true protein digested and absorbed in the small intestine
ESC: ethanol soluble carbohydrates
PA: non-protein nitrogen
PB1: soluble true protein
PB2: intermediately degradable true protein
PB3: slowly degradable true protein
PC: indigestible protein
RUP: rumen undegraded protein
OEB: rumen degraded protein balance
OM: organic matter
